# Role of *SERCA3* in the Prognosis and Immune Function in Pan-Cancer

**DOI:** 10.1155/2022/9359879

**Published:** 2022-11-04

**Authors:** Jiajia Li, Xionghui Li, Hong Huang, Lijian Tao, Chenzi Zhang, Yanyun Xie, Yupeng Jiang

**Affiliations:** ^1^Department of Oncology, The Second Xiangya Hospital, Central South University, Changsha 410008, China; ^2^Department of Nephrology, Xiangya Hospital, Central South University, Changsha 410008, China; ^3^Department of Critical Medicine, Hunan Provincial Hospital of Integrated Traditional Chinese and Western Medicine, Changsha 410008, China; ^4^Guilin Medical University, Guilin 541000, China; ^5^Department of Hematology, Xiangya Hospital, Central South University, Changsha 410008, China

## Abstract

The sarcoendoplasmic reticulum calcium adenosine triphosphatase (ATPase) 3 (*SERCA3*), a member of the SERCA protein family, is located at the endoplasmic reticulum. Its main function is to pump Ca2+ into the endoplasmic reticulum and is involved in maintaining intracellular calcium homeostasis and signal transduction, which are very important factors impacting cancer development and progression. However, the specific role of *SERCA3* in cancer remains unclear. Our study, for the first time, comprehensively analyzed the *SERCA3* expression profile in multiple cancers and its prognostic value in different cancers using bioinformatics. Furthermore, TCGA database was applied to evaluate the certain correlation of *SERCA3* expression with immune modulator genes, immune checkpoints, immune cell infiltration, TMB, and MSI. The results revealed that in many cancers, *SERCA3* expression was markedly decreased, which was related to poor prognosis. Additionally, we noticed that *SERCA3* expression was correlated with TNM classification and WHO cancer stages in some cancer types. The Pearson correlation analysis showed that *SERCA3* expression was closely associated with chemokines, chemokine receptors, MHC, immune activation genes, and immunosuppressive genes. In most cancer types, *SERCA3* expression was also associated with immune checkpoints, including PDCD1 and CTLA-4. Further analysis suggested that *SERCA3* was significantly correlated with CD8+ T cells, and regulatory T cells. Additionally, pan-cancer analysis confirmed that *SERCA3* expression was related to TMB and MSI. In conclusion, these results offer a new insight into the functions and effects of *SERCA3* in pan-cancer, and further provide some basis for considering *SERCA3* as a potential cancer treatment target and biomarker.

## 1. Introduction

Cancer, a major cause of death worldwide, imposed a heavy burden on society [1–4]. Cancer incidence and mortality are exceptionally high. Global cancer cases increased by 19 million in 2020, and nearly 10 million deaths due to cancer were recorded. Furthermore, America cancer cases expected to rise by 1.9 million, and new cancer deaths are expected to reach 60,936 by 2022 [3, 5]. The rapid development of cancer immunotherapy in recent years has improved the prognosis of some cancer patients; however, immune checkpoint inhibitors have not achieved satisfactory results in most cancer cases [6–8]. This may be attributed to the susceptibility of cancer to mutations and drug resistance, which significantly limit cancer screening and treatment [9, 10]. Therefore, identifying new therapeutic targets or biomarkers is important for the early screening and successful treatment of cancer.

The sarcoendoplasmic reticulum calcium adenosine triphosphatase (ATPase) 3 (*SERCA3*) enzyme belongs to the *SERCA* protein family and is found in the endoplasmic reticulum. It pumps calcium ions (Ca^2+^) from the cytoplasm into the endoplasmic reticulum, which is the main calcium-storing organelle. In most cells, it is mainly involved in maintaining homeostasis of endoplasmic reticulum Ca^2+^ and the intracellular Ca^2+^ concentration [11–13]. Being the second messenger of intracellular signal transduction, Ca^2+^ is an important regulator of cellular signaling activities, and intracellular Ca^2+^ disorders can affect gene expression, proliferation, differentiation, and cell death [14–16]. Cumulative evidence suggests that Ca^2+^ signal transduction is crucial for cancer development. The growth, proliferation, invasion, death, and drug resistance of cancer cells are regulated by Ca^2+^ [17–20]. It has been reported that abnormal changes in amplitude of cytoplasmic free Ca^2+^ concentration and duration of Ca^2+^ elevation may promote breast cancer cell proliferation and invasion [17, 21]. The same phenomenon was confirmed in endometrial and colorectal cancers [22, 23].

Intracellular calcium homeostasis is a crucial factor that affects the occurrence and development of cancers. *SERCA3* is one of the most important calcium modulators involved in maintaining intracellular calcium homeostasis by modulating the entry of cytoplasmic calcium into the endoplasmic reticulum. However, no pan-cancer study of *SERCA3* has been reported, and the role of *SERCA3* in pan-cancer remains unknown. Our study elucidated the *SERCA3* expression profile and examined correlations between *SERCA3* expression and cancer prognosis; moreover, the correlation between *SERCA3*, tumor-node-metastasis (TNM) classification, and World Health Organization (WHO) cancer stages was also detected. The relationship between *SERCA3* with immune modulator pathways, immune checkpoints, and immune cell infiltration levels was analyzed. Finally, we examined the correlation of *SERCA3* expression with cancer mutation burden (TMB) and microsatellite instability (MSI). We provided a study of *SERCA3* in pan-cancer, focusing on the role of *SERCA3* in cancer immune functions and the potential mechanisms of cancer immunotherapy.

## 2. Materials and Methods

### 2.1. *SERCA3* Expression in Human Pan-Cancer

The Cancer Genome Atlas (TCGA) pan-cancer database (PANCAN, *N* = 10535, *G* = 60499, year: updated in 2022) was downloaded from the UCSC Cancer Genome Browser (https://xenabrowser.net/), from which *SERCA3* expression data for each cancer type were extracted [24, 25]. Furthermore, we screened data from the Primary Tumor (year: updated in 2022) and Solid Tissue Normal (year: updated in 2022) databases to compare *SERCA3* expression between different cancer types. The final cancer expression data were obtained after eliminating cancer types from less than three sample. All expression data were standardized by log2 conversion. *SERCA3* expression in different cancers was calculated using R software (version 3.6.4) [24]. Additionally, we used the Human Protein Atlas (HPA) database to investigate *SERCA3* expression in normal and cancer tissues in humans.

### 2.2. Association of *SERCA3* Expression with TNM Classification and WHO Cancer Stages

We selected *SERCA3* expression data from TCGA-LAML (year: updated in 2022) and Primary Tumor databases. The final cancer expression data were obtained after eliminating cancer types from less than three sample. Using R software to correlate *SERCA3* expression with TNM classification and WHO cancer stages in various types of cancer. All expression data were standardized via log2 conversion.

### 2.3. Prognostic Analysis

In addition to extraction of data from TCGA-LAML, TCGA-SKCM (year: updated in 2022), and Primary Tumor databases, prognostic data for TCGA within 1 month of follow-up were also obtained from a previously published TCGA prognosis study [26], and pan-cancer data were obtained after eliminating the cancer types with less than 10 samples. Applying hazard ratios (HR) and 95% confidence intervals (CI) to assess overall survival (OS).

### 2.4. Relationship between *SERCA3* Expression and Immune Modulator Pathways and Immune Checkpoints

The *SERCA3* expression data and data on five immune modulator pathways, including chemokines, chemokine receptors, major histocompatibility complex (MHC), immune activation genes, and immunosuppressive genes, were extracted from TCGA. Further, we excavated TCGA-LAML and Primary Tumor data and plotted the Spearman correlation analysis heat map of *SERCA3* expression and five immune modulator pathways.

Moreover, we extracted expression data on two immune checkpoints, including 24 immune checkpoint inhibitors and 36 immune checkpoint stimulators, from a previous study [27]. We screened the cancer samples as follows: TCGA-LAML and Primary Tumor. All expression data were standardized by log2 conversion. The Pearson correlation between *SERCA3* level and two immune checkpoint pathways was calculated.

### 2.5. *SERCA3* Expression and Immune Cell Infiltration

Mapping the obtained *SERCA3* expression data of each cancer type to Gene Symbol, using CIBERSORT [28, 29] in R software IOBR (version 0.99.9) [30]. Immune cell infiltration levels of each cancer type were assessed, the corr.test function of the R software psych (version 2.1.6) was used to calculate the Spearman correlation coefficient.

### 2.6. Association of *SERCA3* Expression with TMB and MSI

*SERCA3* expression and TMB data were extracted from TCGA and Primary Tumor. Downloaded TCGA level 4 simple nucleotide variation data processed by MuTect2 software from GDC [31]. TMB for each cancer type was estimated using the “maftools” R package (version 2.8.05). Subsequently, *SERCA3* expression and TMB data were integrated. The final cancer expression data were obtained after eliminating cancer types from less than three sample. All expression data were standardized via log2 conversion. Spearman's correlation between *SERCA3* expression and TMB was then compared.

Subsequently, we obtained the MSI score of each cancer type from a previous study [32], and the MSI score and *SERCA3* expression data were integrated; less than three samples of cancer types were eliminated, and the final cancer expression data was acquired. All expression data were standardized via log2 conversion. Spearman correlation between *SERCA3* expression and MSI was then compared.

### 2.7. Statistical Analysis

Differential expression of *SERCA3* in various cancer types was evaluated using Student's *t*-test. Kruskal–Wallis test and Mann–Whitney U-test were used to calculate the relationship of *SERCA3* expression with TNM classification and WHO cancer stages. HR and *p*-values for overall survival were assessed using the log-rank test. Spearman correlation and Pearson's correlation were applied to detect the correlation between *SERCA3* expression and immunity. All analyses were performed using R software (IOBR, psych, and maftools). *p* ≤ 0.05 was considered a statistically significant difference.

## 3. Results

### 3.1. *SERCA3* Expression in Human Pan-Cancer

**Figure 1:**
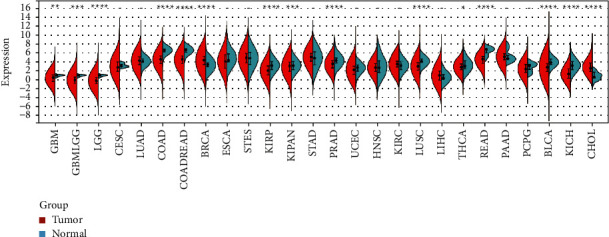
*SERCA3* expression in human pan-cancer. The expression of *SERCA3* in different cancer types were compared in 26 cancer types based on the Solid Tissue Normal, Primary Blood Derived Cancer- Peripheral Blood, Primary Tumor database. ^*∗*^*p* ≤ 0.05; ^*∗∗*^*p* < 0.01; ^*∗∗∗*^*p* < 0.001 and ^*∗∗∗∗*^*p* < 0.0001.

**Figure 2:**
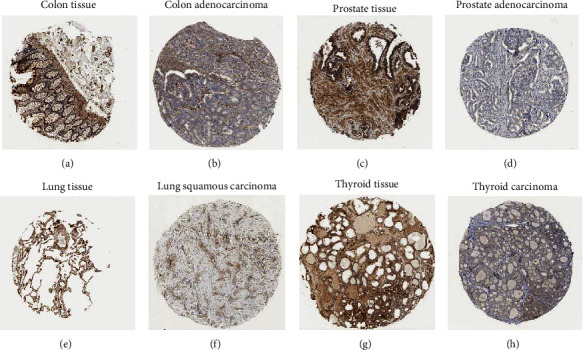
The IHC of *SERCA3* between human normal tissues and cancer tissues. *SERCA3* expression levels in human normal tissues and cancer tissues from The Human Protein Atlas database. ((a), (c), (e), (g)) normal colon, prostate, lung, thyroid. ((b), (d), (f), (h)) colon adenocarcinoma, prostate adenocarcinoma, lung squamous carcinoma, thyroid carcinoma.

We calculated *SERCA3* expression in various cancer types based on TCGA database. The results showed inconsistent expression of *SERCA3* in different types of cancer; it had significantly low expression in 13 cancers, including GBM, GBMLGG, LGG, COAD, COADREAD, KIRP, KIPAN, PRAD, LUSC, THCA, READ, BLCA, and KICH. Contrastingly, two cancers, including BRCA and CHOL, showed significantly high *SERCA3* expression (Figure 1). Immunohistochemistry (IHC) of *SERCA3* in COAD, PRAD, LUSC, and THCA supported this view (Figure 2). These cancer abbreviations are defined in Supplement 1.

### 3.2. Association of *SERCA3* Expression with TNM Classification and WHO Cancer Stages

**Figure 3:**
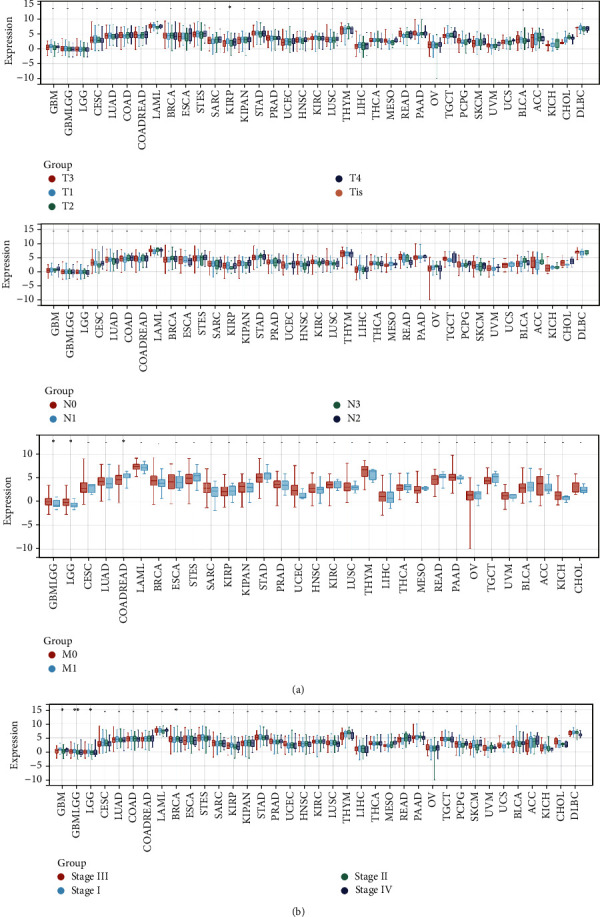
Pan-Cancer Analysis of the Association between *SERCA3* Expression and TNM classification and WHO cancer stages (a) The correlations between *SERCA3* expression and TNM classification. (b) The correlations between *SERCA3* expression and WHO cancer stages.  ^*∗*^*p* ≤ 0.05 and  ^*∗∗*^*p* < 0.01.

To understand the association of *SERCA3* expression with TNM classification and WHO cancer stage, we measured *SERCA3* expression among the different TNM classification. Strong association of *SERCA3* expression with TNM classification was found in KIRP (*p*=0.01), GBMLGG (*p*=0.02), LGG (*p*=0.02), and COADREAD (*p*=0.05) (Figure 3(a)). Subsequently, the expression of *SERCA3* in the WHO cancer stages was assessed based on the Union for International Cancer Control definition. *SERCA3* expression was downregulated in some advanced-stage cancers, including GBMLGG (*p*=1.5*e* − 3), BRCA (*p*=0.05), LGG (*p*=0.04), and GBM (*p*=0.02) (Figure 3(b)).

### 3.3. Prognostic Analysis of *SERCA3* Expression

**Figure 4:**
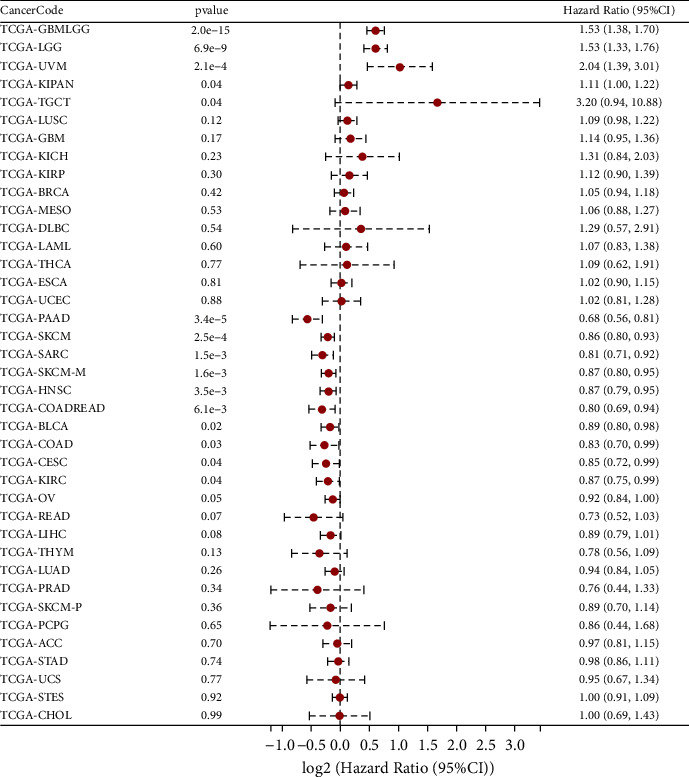
Prognostic analysis of *SERCA3* expression. The forest map shows the influence of *SERCA3* expression on overall survival (OS) evaluated by Cox proportional hazard regression model.

The relevance between the expression of *SERCA3* and the OS in cancer patients was evaluated. *SERCA3* is a protective factor in most cancers, HR and 95%CI for cancers were PAAD (0.68, 0.56–0.81), CESC (0.85, 0.72–0.99), SKCM (0.86, 0.80–0.93), SARC (0.81, 0.71–0.92), BLCA (0.89, 0.80–0.98), SKCM-M (0.87, 0.80–0.95), COADREAD (0.80, 0.69–0.94), HNSC (0.87, 0.79–0.95), KIRC (0.87, 0.75–0.99), COAD (0.83, 0.70–0.99), OV (0.92, 0.84–1.00), while *SERCA3* is an adverse factor in KIPAN (1.11, 1.00–1.22), GBMLGG (1.53, 1.38–1.70), TGCT (3.20, 0.94–10.88), UVM (2.04, 1.39–3.01), LGG (1.53, 1.33–1.76). The pan-cancer results were found using cox regression analysis (Figure 4).

### 3.4. Relationship between *SERCA3* Expression and Immune Modulator Pathways and Immune Checkpoints

**Figure 5:**
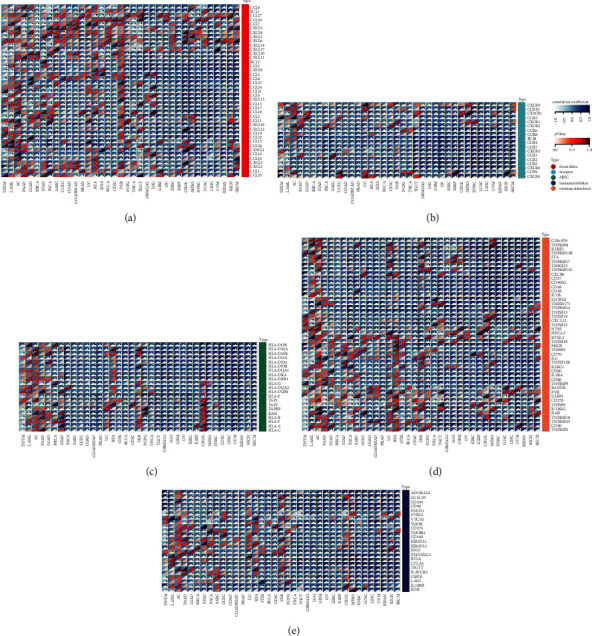
Pan-Cancer Analysis of the *SERCA3* Expression in Relation to Immune Modulator Pathways (a) The heatmap shows the correlation between *SERCA3* expression and chemokines. (b) The heatmap shows the correlation between *SERCA3* expression and chemokine receptors. (c) The heatmap shows the correlation between *SERCA3* expression and MHC. (d) The heatmap shows the correlation between *SERCA3* expression and immune activation genes. (e) The heatmap shows the correlation between *SERCA3* expression and immunosuppressive genes. For each pair, the left top triangle is colored to represent the Spearman correlation coefficient; the right bottom one is colored to indicate the *p*-value.  ^*∗*^*p* ≤ 0.05.

Based on TCGA database, we analyzed the connection between *SERCA3* expression and the five immune modulator pathways. The heat map revealed that *SERCA3* expression was closely correlated with the level of chemokines and chemokine receptors, such as *CCL5*, *CCL17*, *CCL22*, *CCR4*, and *CCR5* (Figures 5(a) and 5(b)). Furthermore, *SERCA3* expression was closely correlated with MHC, immune activation genes, and immunosuppressive genes such as *HLA-DRB1*, *HLA-E*, *PDCD1 (PD-1)*, *TGF-B1*, *CTLA-4*, *TIGIT*, and *ICOS* in most cancer types (Figures 5(c)–5(e)).

**Figure 6:**
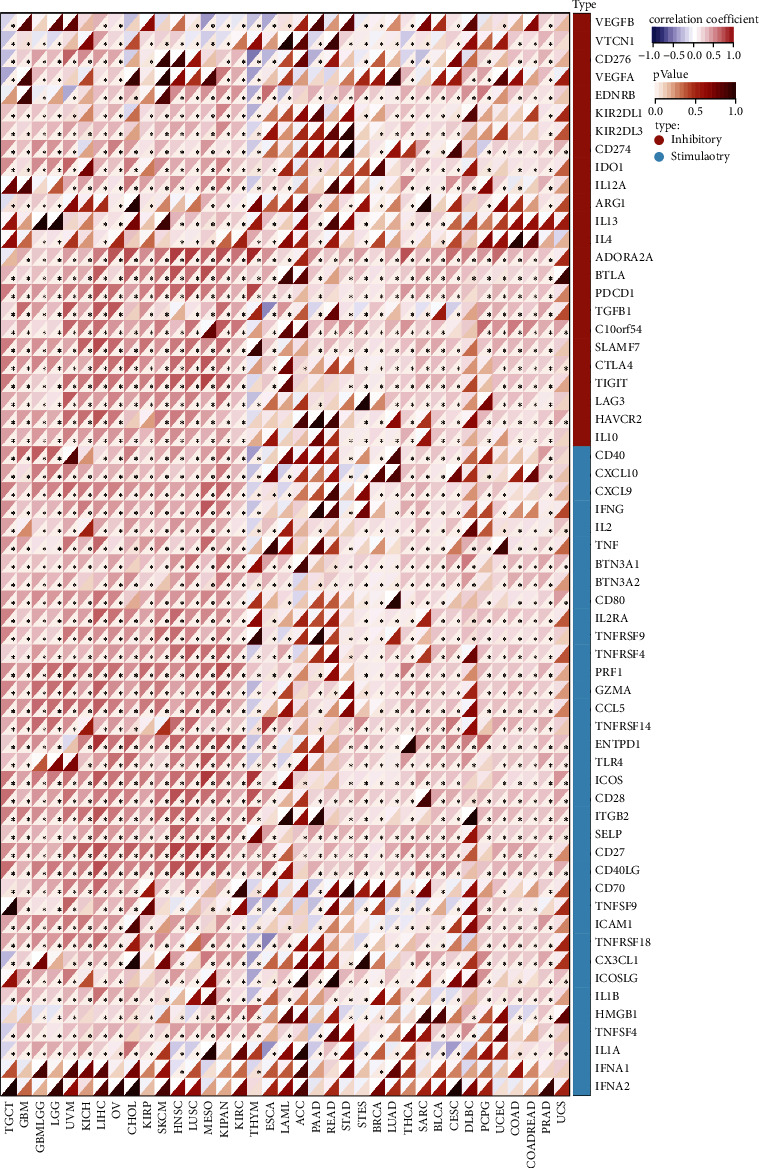
Pan-cancer analysis of the *SERCA3* expression in relation to immune checkpoints. The heatmap shows the correlation between *SERCA3* expression and immune checkpoints. For each pair, the left top triangle is colored to represent the Spearman correlation coefficient; the right bottom one is colored to indicate the *p*-value.  ^*∗*^*p* ≤ 0.05.

Immunotherapy is increasingly becoming an important means of cancer treatment, the application of immune checkpoint inhibitors has improved the prognosis of some cancer patients [33, 34]. Therefore, we collected the expression data of 60 common immune checkpoints [27], using Pearson's correlation analyzed the relationship between *SERCA3* expression and immune checkpoints. Our results suggested that in most types of cancer, *SERCA3* expression was distinctly related to immune checkpoints, such as *TLR4*, *ICOS*, *CTLA-4*, *PDCD1*, and *CD27* (Figure 6).

### 3.5. Immune Cell Infiltration Analysis

**Figure 7:**
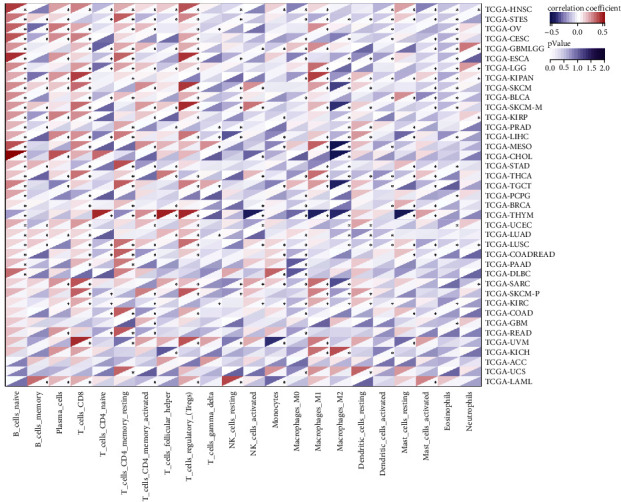
Immune cell infiltration analysis. The heatmap shows the correlation between *SERCA3* expression and immune cell infiltration levels. For each pair, the left top triangle is colored to represent the Spearman correlation coefficient; the right bottom one is colored to indicate the *p*-value.  ^*∗*^*p* ≤ 0.05.

The abundances of 22 immune cells were calculated using CIBERSORT, the relationship between *SERCA3* expression and immune cell infiltration levels in different cancer types was analyzed. We noticed that the abundance of many immune cells was correlated with *SERCA3* expression. *SERCA3* expression was positively connected with CD8^+^ T cells, regulatory T (Treg) cells, M1 macrophages, and naïve B cells, while negatively correlated with M0 macrophages, M2 macrophages, and eosinophils (Figure 7).

### 3.6. Association of *SERCA3* Expression with TMB and MSI

**Figure 8:**
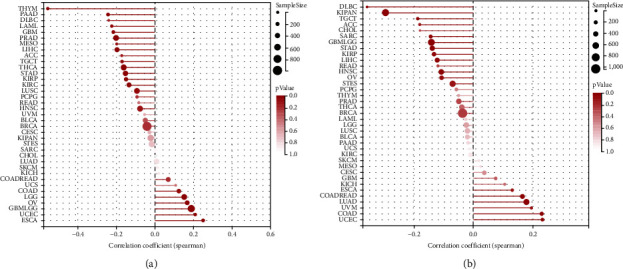
The Relationship between *SERCA3* Expression and TMB and MSI (a) Map exhibits the correlations between *SERCA3* expression and TMB. (b) Map exhibits the correlations between *SERCA3* expression and MSI. Point size represents sample size, the larger the point, the larger the samples size; red color depth represents *p* value, the deeper the color, the smaller the *p* value.

TMB and MSI affect the sensitivity of immunotherapy and prognosis. The current study analyzed whether there is a correlation between *SERCA3* expression and TMB and MSI in various cancers. From the analysis results it seems that *SERCA3* expression was positively correlated with TMB in some cancers. A *p*-value for these cancers were UCEC (0.0052), LGG (0.0006), OV (0.0035), COAD (0.0360), ESCA (0.0007), and GBMLGG (<0.0001), while it was negatively associated with TMB in LIHC (0.0002), TGCT (0.0431), PAAD (0.0016), PRAD (<0.0001), LAML (0.0131), GBM (0.0089), THCA (0.0004), STAD (0.0018), THYM (7.87e-11), KIRP (0.0126), LUSC (0.0403), and KIRC (0.0137) (Figure 8(a)). Moreover, expression of *SERCA3* was positively associated with MSI in some cancers. A *p*-value for these cancers were COADREAD (0.0014), LUAD (<0.0001), COAD (<0.0001), and UCEC (0.0017), and was negatively correlated with MSI in TGCT (0.0224), STAD (0.005), LIHC (0.0202), DLBC (0.0136), KIPAN (2.16e-15), GBMLGG (0.0003), SARC (0.0231), HNSC (0.0161), and KIRP (0.0251) (Figure 8(b)).

## 4. Discussion

Calcium-dependent cell signal transduction was involved in a variety of life activities including proliferation, differentiation, secretion, and death [12]. Maintaining Ca^2+^ homeostasis is crucial for protein storage and transport, signal transduction, and various cellular activities [11]. Abnormal changes in intracellular Ca^2+^ levels have been reported to affect cancer progression [21, 22, 35, 36]. However, in cancer, the role of *SERCA3*, a protein that maintains Ca^2+^ homeostasis in the cytoplasm and endoplasmic reticulum, remains unknown. In this study, the pan-cancer analysis revealed an association between *SERCA3* expression and cancer prognosis, immunoregulatory genes, immune infiltration, and mutations.

We found that *SERCA3* expression varied among different cancer types. *SERCA3* was expressed at low levels in 13 types of cancers, including GBM, GBMLGG, LGG, COAD, COADREAD, KIRP, KIPAN, PRAD, LUSC, THCA, READ, BLCA, and KICH. Comparative analyses revealed high *SERCA3* expression in two cancer types, including BRCA and CHOL. Moreover, *SERCA3* expression is association with WHO cancer stages and TNM classification in a few types of cancer. For instance, the expression of *SERCA3* is different for WHO cancer stages of GBM, GBMLGG, LGG, and BRCA. Furthermore, the expression of *SERCA3* is related to metastasis stages of GBMLGG, LGG, and COADREAD. Cox regression analysis showed that *SERCA3* is a protective factor against some cancers, including PAAD, SKCM, SARC, SKCM-M, HNSC, COADREAD, BLCA, COAD, CESC, KIRC, and OV. However, it acts also as a risk factor for GBMLGG, LGG, UVM, KIPAN, and TGCT. These results indicated that *SERCA3* has a low level of expression in most cancers compared with its expression in normal tissues and plays a protective role in most cancer types.

Analysis of the results of the TCGA database revealed that the expression of *SERCA3* was correlated with the chemokine receptors *CCR4*, which plays a significant role in immune regulation and is regarded as a potential therapeutic target in bronchial asthma. *CCR4* is also highly expressed in adult T-cell leukemia/lymphoma (ATLL) and cutaneous T-cell lymphoma (CTCLs) [37]. Li et al. showed that overexpression of *CCR4* mediates the chemotactic response of breast cancer cells to *CCL17* and accelerates the growth and metastasis of breast cancer [38]. Our results found a correlation between the level of *SERCA3* and immune-activating and immunosuppressive genes, including *PDCD1 (PD-1)*, *CTLA-4*, *TIGIT*, and *ICOS*. By analyzing the correlation between *SERCA3* expression and immune checkpoints we found that *SERCA3* expression was related to immune checkpoints, including *CTLA-4*, *PDCD1*, and *ICOS* in most types of cancer. *PDCD1* and *CTLA-4* antibodies, which are immune checkpoint inhibitors, have been approved for the treatment of cancers including non-small cell lung cancer (NSCLC) and melanoma, and have improved the prognosis of patients with these cancers [39, 40]. These results proved that *SERCA3* might partially affect immune checkpoints.

The tumor microenvironment (TME) is pivotal in regulating cancer progression and can predict treatment outcomes [41–43]. The composition of the TME is complex and includes vascular vessels, immune infiltrates, fibroblasts, and the extracellular matrix [44–46]. The immune cells, an important part of the TME, show an apparent impact on cancer development [46, 47]. Investigating the association of *SERCA3* expression and levels of immune cell, we detected that *SERCA3* expression was positively associated with M1 macrophages and CD8^+^ T cells levels, whereas it showed a negative correlation with the levels of M0 and M2 macrophages. Cytotoxic CD8^+^ T cells are the main immune cells against pathogens and neoplastic cells. The cancer immunotherapy partially strengthens CD8+ T cell activity leading to the reduced escape of cancer cells from the immune system and then establishing durable and efficient anti-tumor immunity [48, 49]. *SERCA3* may play a protective role in most cancers by increasing T cell infiltration. Previous research reported that an increased M2/M1 macrophage ratio promotes cancer progression [50]. *SERCA3* expression was positively correlation with M1 macrophage levels while negatively correlation with M2 macrophage levels, further providing a basis for the protective role of *SERCA3* in most cancer types. These results suggest that *SERCA3* may interfere with the prognosis of various cancers by regulating the expression of multiple immune cells.

Finally, we assessed the correlation among *SERCA3* expression, TMB, and MSI. The more somatic mutations in tumors, the newer antigens that may form, and TMB can be used to evaluate the number of new tumor antigen loads [51]. MSI is an indicator of DNA mismatch repair (MMR) defects. TMB and MSI were used as biomarkers to predict the efficacy of immune checkpoint blockade (ICB) [52, 53]. By pan-cancer analysis we found that *SERCA3* expression correlated with TMB and MSI, providing evidence for *SERCA3* as a potential predictor of ICB therapy.

However, our study had some limitations. First, it was based on bioinformatics and different databases; methods of generating data may have impacted the results. Second, TCGA database lacks data on immunotherapy; hence, we cannot further analyze the indications for immunotherapy. Overall, our study systematically analyzed the association of *SERCA3* expression with prognosis, immune modulator genes, immune checkpoints, immune cell infiltration, TMB, and MSI, which can provide information to further understand the role of *SERCA3* in cancers and its relationship with the immune responses. It also provides a basis for considering *SERCA3* as a potential cancer treatment target and biomarker. A potential challenge in the future will involve the development of new therapeutic methods related to the specific targeting of *SERCA3* to limit the development and progression of cancer.

## 5. Conclusions

This research revealed that *SERCA3* expression was significantly decreased in most types of cancer, cancer patients with reduced *SERCA3* expression tend to have a poor prognosis. Moreover, we analyzed the correlation of *SERCA3* expression with immune regulatory gene expression, immune checkpoints, immune cell infiltration, TMB, and MSI. We speculated that *SERCA3* might affect cancer progression by regulating the TME, especially immune cells. These results provide new ideas for the function and role of *SERCA3* in pan-cancer and provide a theoretical basis for considering *SERCA3* as a potential cancer treatment target and biomarker.

## Data Availability

The data used in this study can be found in the relevant literature, The Human Protein Atlas (HPA: https://www.proteinatlas.org), UCSC (https://xenabrowser.net), GDC (https://portal.gdc.cancer.gov), TCGA-SKCM, Solid Tissue Normal, Primary Blood Derived cancer-peripheral Blood and Primary Tumor.

## Supplementary Materials

Supplementary Materials Supplement 1 Abbreviations of nouns.
